# PCR-Free Detection of Genetically Modified Organisms Using Magnetic Capture Technology and Fluorescence Cross-Correlation Spectroscopy

**DOI:** 10.1371/journal.pone.0008074

**Published:** 2009-11-26

**Authors:** Xiaoming Zhou, Da Xing, Yonghong Tang, Wei R. Chen

**Affiliations:** 1 MOE Key Laboratory of Laser Life Science & Institute of Laser Life Science, College of Biophotonics, South China Normal University, Guangzhou, China; 2 Department of Engineering and Physics College of Mathematics and Science, University of Central Oklahoma, Edmond, Oklahoma, United States of America; University of Portsmouth, United Kingdom

## Abstract

The safety of genetically modified organisms (GMOs) has attracted much attention recently. Polymerase chain reaction (PCR) amplification is a common method used in the identification of GMOs. However, a major disadvantage of PCR is the potential amplification of non-target DNA, causing false-positive identification. Thus, there remains a need for a simple, reliable and ultrasensitive method to identify and quantify GMO in crops. This report is to introduce a magnetic bead-based PCR-free method for rapid detection of GMOs using dual-color fluorescence cross-correlation spectroscopy (FCCS). The cauliflower mosaic virus 35S (CaMV35S) promoter commonly used in transgenic products was targeted. CaMV35S target was captured by a biotin-labeled nucleic acid probe and then purified using streptavidin-coated magnetic beads through biotin-streptavidin linkage. The purified target DNA fragment was hybridized with two nucleic acid probes labeled respectively by Rhodamine Green and Cy5 dyes. Finally, FCCS was used to detect and quantify the target DNA fragment through simultaneously detecting the fluorescence emissions from the two dyes. In our study, GMOs in genetically engineered soybeans and tomatoes were detected, using the magnetic bead-based PCR-free FCCS method. A detection limit of 50 pM GMOs target was achieved and PCR-free detection of GMOs from 5 µg genomic DNA with magnetic capture technology was accomplished. Also, the accuracy of GMO determination by the FCCS method is verified by spectrophotometry at 260 nm using PCR amplified target DNA fragment from GM tomato. The new method is rapid and effective as demonstrated in our experiments and can be easily extended to high-throughput and automatic screening format. We believe that the new magnetic bead-assisted FCCS detection technique will be a useful tool for PCR-free GMOs identification and other specific nucleic acids.

## Introduction

Genetically modified organisms (GMOs) or transgenic crops have been developed in an attempt to improve food quality and solve problems associated with commercial agriculture, including disease and weed management [1]. Consumer concerns about the safety of GMOs has prompted the development in GMOs food labeling legislation. A threshold for affirmative GMOs labeling has been adopted in many countries [2]–[3]. Demands for testing GMO foods and interests for development of reliable GMOs detection methods have been increased dramatically. Currently, the two most prevalent approaches for GMO detection are DNA-based PCR and antibody-based immunoassays [4]–[5]. However, protein-based assays are not suitable for processed food and DNA-based PCR suffers from the problems of amplification related errors. To overcome these limitations, attempts have been made to directly identify GMOs from unamplified genomic DNA recently [6]–[7].

Recent developments in laser-based detection of single fluorescent molecules have made possible the implementation of sensitive techniques for biochemical analysis. One of the most prominent single-molecule detection techniques is fluorescence correlation spectroscopy (FCS) [8]–[11]. FCS detects fluorescence fluctuations caused by the Brownian motion of a single molecule diffusing across a volume focused by a laser beam. Since the binding of a relatively small, fluorescence-labeled molecule to a larger ligand results in a change of diffusion time, FCS can quantify interactions between the molecules at extremely low concentrations and in small volumes [8], [12]. FCS has been used to quantitatively analyze pathogen genomic DNA amplified by PCR, with high sensitivity [13]. However, in auto-correlation experiments using FCS, in order to distinguish two different species of molecules, their diffusion times should be at least 1.6-fold apart, which means that the size difference of molecules should be 5-fold or more [14]. Therefore, FCS has mainly been used for the molecular reactions between one small labeled ligand and a relatively large nonfluorescent counterpart within the measurement volume [15]–[16].

Dual-color fluorescence cross-correlation spectroscopy (FCCS), realized experimentally first by Schwille et. al. [17], is an extended version of FCS. In the dual-color cross-correlation system, two spectrally distinct fluorophores in the same volume are independently excited by two different excitation sources, and simultaneous fluctuations of the fluorescence signals in the two color channels indicate the presence of tight chemical or physical linkages between the fluorophores. In fact, there is only one prerequisite for FCCS in principle: the two differently labeled partners have to move independently at first and then bind together during the detection process. The system allows for probing of extremely low fluorophore concentrations with a separation-free format.

FCCS has become a valuable tool for interaction studies in living cells [18]. FCCS can also be used for detecting DNA sequences hybridized with two complementary gene probes labeled with two different fluorescent dyes, ideal for nucleic acid and enzyme assays. For example, FCCS technique has been successfully applied to DNA enzymatic assay [19]–[20], gene expression analysis [21]–[23], and allele distinction [24] at single molecule level.

Inspired by these FCCS applications, we hypothesize that this technique can be extended to direct identify GMOs from unamplified samples. However, the extremely high complexity of the unamplified target and sample media, such as fluorescence interference and enzyme interference, are often limiting factors for direct GMO identification. One strategy used in conventional southern blot for reduction in complexity is an electrophoresis based method. This procedure is time consuming, typically requiring the genomic DNA sample to be cleaved with a restriction enzyme, size separated by gel electrophoresis, and recovered from the gel to a nitrocellulose filter for subsequent hybridization and detection. Another challenge is that the genomic DNA consists of long chains of double-stranded DNA (dsDNA). Therefore, to achieve effective hybridization, problems such as cularization of the genomic DNA and supercoiling emergence during the heat denaturation step should be avoided [6], [25]. To solve these problems, it is highly desirable for detection probes to hybrid with the target in single-strand format and in clean environments. One strategy with increasing popularity in recent years is the use of surface-functionalized magnetic micro- or nano-particles to selectively bind and preconcentrate low-abundance target analytes (DNA, bacteria, protein) and to discard the sample matrix prior to the detection procedures. Magnetic beads have been proven valuable in immune and nucleic acid assays with improved sensitivity and selectivity [26]–[28]. The possibility of magnetically confining and concentrating target analytes offers great potential for the detection of minute amounts of target DNA. Combining such a magnetic capture technique with FCCS could theoretically revolutionize the detection of low-level target DNA, particularly for the identification of unamplified GMOs. To the best of our knowledge, the feasibility of such a combination has not been demonstrated experimentally.

## Materials and Methods

### Fluorescence Correlation Spectroscopy (FCS)

FCS can be used to analyze fluorescence intensity fluctuations originated from fluorescence-labeled molecules diffusing through a small confocal detection volume [8], [29]. Correlation of the intensity fluctuations over time yields an auto-correlation function, *G*(τ), which provides two important analytical parameters: the average number of molecules in the detection volume and the translational diffusion time of the individual molecules. The experimental auto-correlation can be described by a one-component model [30]:
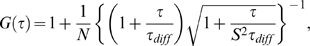
(1)where *τ* is the correlation time, *N* is the average number of fluorescently labeled molecules in the confocal volume, the structural parameter *S* is the ratio of *z* to *ω*, the half axes of the confocal volume, and τ*_diff_* is the diffusion time of fluorescently labeled molecules, which is defined as:
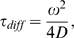
(2)where *D* is the diffusion coefficient. The effective confocal volume, *V_eff_* is defined as:
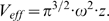
(3)


### Fluorescence Cross-Correlation Spectroscopy (FCCS)

In dual-color FCCS, the gene probes labeled by two different fluorescent dyes and hybridized with the target DNA fragment can be excited simultaneously by two different laser beams and the fluorescence signals from the dyes can be detected in two separate channels. In addition to the auto-correlation functions, the cross-correlation function between the fluorescence intensities of green (*g*) and red (*r*) dyes, *G_gr_*(τ), can be determined.

The cross-correlation function can be described as [30]:
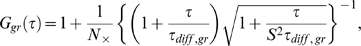
(4)where 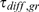 is the diffusion time of the DNA molecules doubly labeled with green and red dyes and 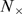 is the inverse amplitude of the cross-correlation function:
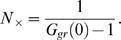
(5)


Assuming that there is no cross-talking between the two detection channels, the number of doubly labeled molecules in the detection volume, *N_gr_*, can be determined by [30]:
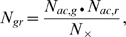
(6)where *N_ac,g_* and *N_ac,r_* are the numbers of the singly labeled molecules in the detection volume obtained from the auto-correlation functions according to Equation (1) in the green and red detection channels, respectively. For FCCS using a 488-nm line and a 633-nm line, the effective detection volume can be calculated by
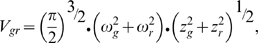
(7)where *ω_g_* and *z_g_* denote the half axes of the confocal volume of the 488-nm line, and *ω_r_* and *z_r_* denote that of the 633-nm line.

### Reagents

β-Mercaptoethanol was purchased from AMRESCO, Netherlands. Streptavidin-coated magnetic beads (2.8 µm diameter) were product of Dynal Biotech (Lake Success, NY, USA). Restriction endonucleases (EcoR V and Hinf I) and the gene probes labeled with biotin, Rhodamine Green, and Cy5 dyes were purchased from TaKaRa Biotechnology Co. Ltd, (Dalian. China). The excitation and emission peaks of RG are 502 nm and 527 nm, and those of Cy5 are 649 nm and 660 nm, respectively. GM and non-GM soybeans and tomatoes were from South China Agricultural University (Guangzhou, China).

### DNA Extraction

The cetyltrimethyl ammonium bromide (CTAB) method for sample extraction and purification was used in this study [5]. The samples were minced with sterile surgical blades and the dry powder was moistened with a threefold amount of water. The DNA samples, with or without GM components, were extracted using the CTAB method, precipitated, treated with chloroform, and precipitated with isopropanol to obtain a purified DNA matrix.

### Cleavage of the Genomic DNA with Two Restriction Enzymes

The functioned sequences of EcoR V and Hinf I are 5′-GATATC-3′ and 5′-GANTC-3′, respectively. They were used to cleave a 211 bp target DNA fragment from the CaMV35S promoter in the GM samples. The reactions of EcoR V and Hinf I were carried out in a buffer containing 100 mM NaCl, 50 mM Tris-HCl (pH 7.5), 10 mM MgCl_2_, and 1 mM DTT; at 37°C for 1–4 hours. The cleaved product was precipitated with ethanol and then resuspended in TE buffer (pH 7.4).

### Purification of the Target DNA Fragment by Streptavidin-Coated Magnetic Beads

We designed a biotin-labeled probe for specific capture of the 211 bp target DNA fragment of CaMV35S. The biotin-labeled probe was 5′-Bio-TATCACATCAATCCACTTGCTTTGAAGA-3′, which is complementary to and can specifically hybridize with the target DNA fragment. The cleaved product in TE buffer (pH 7.4) and excess biotin-labeled probe were placed in a water bath, and hybridization was carried out at 94°C for ten minutes and then at 50°C for an hour. After hybridization, the solution was mixed with 20 µL of streptavidin-coated magnetic beads and vigorously stirred at 37°C for 60 min, to form a biotin-streptavidin linkage. A magnetic separator was used to concentrate unreacted magnetic beads and the magnetic beads that have captured target DNA fragment. These beads were washed with TE buffer to remove any unexpected DNA. Then, the magnetic beads were resuspended in NANOpure (18 MW) water in 40°C to dehybridize target DNA fragment from the biotin-labeled probe on the magnetic beads surface. The supernatant fluid containing purified 211 bp target DNA fragment was then easily separated and collected from the beads using the magnetic separator.

### Hybridization of Target DNA Fragment with the Two Dye-Labeled Probes

We designed two gene probes for optimal sensitivity and accuracy of target DNA detection. The two dye-labeled gene probes were 5′ Rhodamine Green–TTTCCACGATGCTCCTCGTGGGTGGG 3′ and 5′ CGGCAGAGGCATCTTCAACGATGGCC–Cy5 3′. They are complementary to and can hybridize specifically with the 211 bp target DNA fragment of CaMV35S. The purified 211 bp target DNA fragment and excess Rhodamine Green-labeled and Cy5-labeled gene probes were placed in a water bath, and hybridization was carried out at 94°C for five minutes and then at 50°C for an hour.

### Fluorescence Correlation Spectrometer and Measurement of Auto- and Cross-Correlation Functions

**Figure 1:**
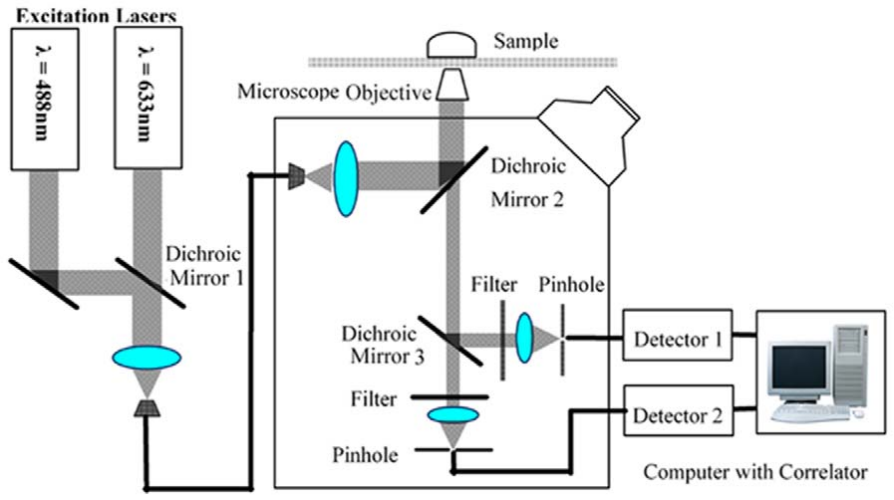
Schematic of the fluorescence correlation spectrometer. An argon-ion laser (488 nm) and a helium-neon laser (633 nm) are used for the excitation of the RG dye and the Cy5 dye in the DNA sample through Dichroic Mirrors 1 and 2. The sample is contained in a chamber with a cover glass and is placed on the stage of an inverted microscope. Fluorescent light from the sample is collected by a high numerical aperture objective lens and transmitted by Dichroic Mirror 2. After passing through Dichroic Mirror 3, the residual laser excitation light and Raman scattered light are removed by additional band-pass filters. Green fluorescence between 505 nm and 550 nm is recorded by Detector 1 and red fluorescence above 650 nm is recorded by Detector 2. A computer was used to control the instrument and to obtain the auto- and cross-correlation functions of the fluorescent intensities, using the counted photoelectron pulses.

The DNA samples were measured with a fluorescence correlation spectrometer (ConfoCor2, Carl Zeiss, Jena, Germany), which is schematically shown in Figure 1 and has been described previously [31]–[32]. The instrument implements the optical principle of confocal arrangements [33], i.e., the focal plane is inside the sample and the pinhole is placed at the optical conjugate point. An argon-ion laser (488 nm) and a helium-neon laser (633 nm) were used for the excitation of the RG dye and the Cy5 dye, respectively. The sample was contained in a chamber with a cover glass on top, and was then placed on the stage of an inverted microscope (Axiovert 200 M; Zeiss). The excitation light was reflected by a dichroic mirror and focused on the focal plane within the sample by an objective (C-Apochromat ×60/1.2). The emitted fluorescent light was collected by a high numerical aperture objective and passed through the dichroic mirror, which reflected the excitation light toward the objective and transmitted the fluorescent light. The residual laser excitation light and Raman scattered light were removed by additional band-pass filters. After passing through another dichroic mirror, the green and red fluorescent lights were focused on actively quenched avalanche photodiodes (photon counting mode; EG&G, Massachusetts, USA) and recorded by Detectors 1 and 2, respectively. Ten samples were prepared for each specimen and each sample was measured ten times (10 seconds each measurement) and the results were averaged. The data from the ten samples were averaged and the auto- and cross-correlation functions were obtained according to Equations (1) and (4).

## Results and Discussion

**Figure 2:**
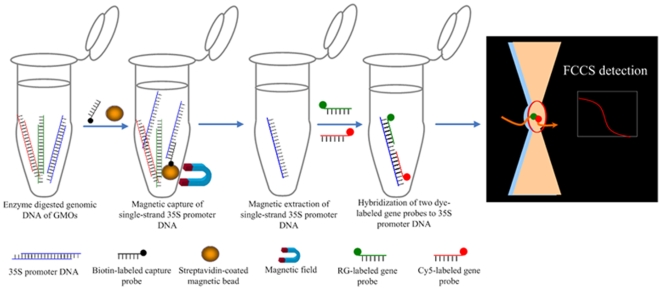
Proposed methodology for PCR-free identification of GMOs by magnetic capture-FCCS. Genomic DNA is isolated from GMOs and then fragmented. Biotin-labeled DNA is hybridized with the 35S promoter region and streptavidin coated-magnetic beads are used to capture the targets from the sample and then washed. The single strand targets are released and hybridized with two fluorophore labeled probes for FCCS detection.

Herein, we report a convenient, simple sample clean up assay, combining with the sensitive FCCS technique, to directly identify GMOs from unamplified samples by targeting CaMV35S, the 35S subunit of the cauliflower mosaic virus, which is a common promoter used in most available transgenic products. In this novel method, genomic DNA was extracted using standard CTAB method and fragmented by restriction endonucleases (EcoR V: 5′-GATATC-3′ and Hinf I: 5′-GANTC-3′). The resultant 211 bp target DNA fragment of CaMV35S was captured by a biotin-labeled nucleic acid probe (5′-Bio-TATCACATCAATCCACTTGCTTTGAAGA-3′). Subsequently, streptavidin-coated magnetic beads were used for purifying the target through biotin-streptavidin linkage. Then, the purified target DNA fragment of CaMV35S was hybridized with two dye-labeled gene probes (5′ Rhodamine Green–TTTCCACGATGCTCCTCGTGGGTGGG 3′ and 5′CGGCAGAGGCATCTTCAACGATGGCC–Cy5 3′) which were specifically complementary to the target in the 35S sequence. Rhodamine Green and Cy5 labels were chosen because of their distinctive absorption and excitation spectra and their well-separated emission profiles, as well as because of their low energy transfer with the DNA target, as demonstrated in the literatures [17], [19]. Finally, FCCS was used to detect the target fragment by simultaneously detecting the fluorescence emissions from the two dyes. If the two spectrally different probes were bound to the same DNA target fragment, their fluorescence signals would reflect similar characteristics, signifying the presence of a 35S target molecule. The doubly bound DNA fragment could be observed in the amplitude of cross-correlation directly, since the correlation times of the single probes were different from that of the target DNA fragment hybridized with both dye labeled probes. The strategy of this method is shown in Figure 2.

The confocal volume of the excitation laser light in FCCS must be determined in order to quantitatively determine the concentration and the kinetics parameters of the sample. To determine the effective volume (*V_gr_*) for the doubly labeled CaMV35S, the confocal volume of the individual excitation laser light (488 and 633 nm) in FCCS must be known. In our experiments, Rhodamine Green (RG) and Cy5 dyes, whose diffusion coefficients in water were reported as 2.8×10^−10^ m^2^/sec and 3.16×10^−10^ m^2^/sec, were chosen to calibrate the confocal volumes of the 488-nm and 633-nm laser beams, respectively [30]. We obtained the fluorescence auto-correlation functions of RG and Cy5 in 1 nM solutions (data not shown), from which the diffusion time and the structural parameter of the confocal volume were determined for RG and Cy5, using the one-component model [30]. Similarly, the half axes of the confocal volume, *ω* and *z*, as well as the effective volume, were calculated for the 488-nm and the 633-nm laser beams. Typically, volumes of 0.159 and 0.567 fL were found for the green and red channel, respectively. To maximize the overlap of the two detection volumes, pinhole alignment was performed as described by Kirsten et al. [34]. These results, summarized in Table 1, led to the determination of *V_gr_* as 0.3458 fL.

**Table 1:** Calibration results for the 488-nm and the 633-nm lines using Rhodamine green and Cy5 dyes by fluorescence correlation spectroscopy.

Dye	Laser line (nm)	τ*_diff_* § (µsec)	*z* § (µm)	ω§ (µm)	S§	*V*_eff_ § (fL)
Rhodamine Green	488	22	1.162	0.157	7.4	0.159
Cy5	633	38	2.124	0.219	9.7	0.567

§ τ*_diff_*, *z*, ω, *S*, and *V*_eff_ are defined in Equations (1), (2), and (3) (see text).

**Figure 3:**
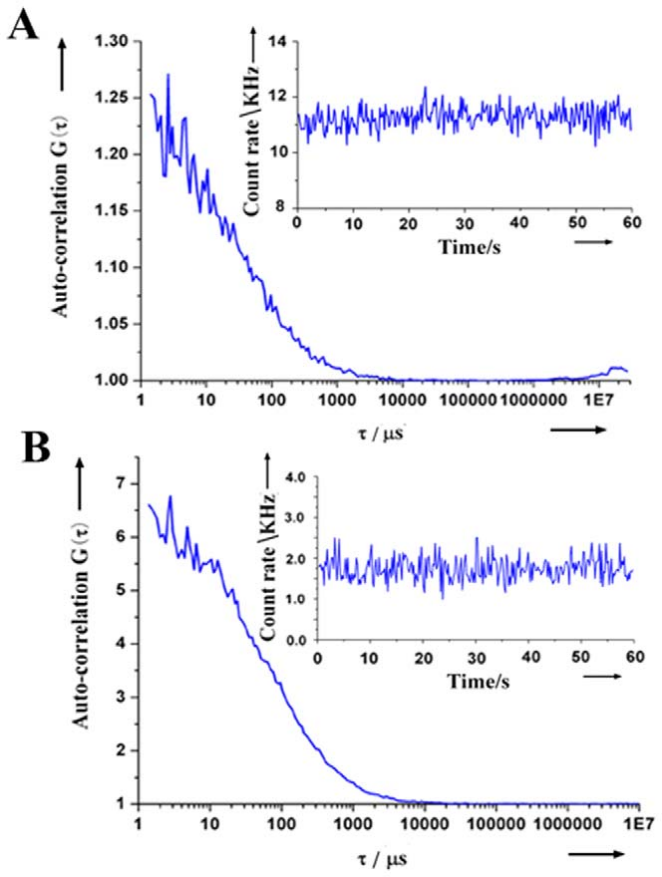
Auto-correlation functions and count rates (insets) of dye-labeled gene probes measured by FCS; (A) Rhodamine Green-labeled gene probes; (B) Cy5-labeled gene probes. The measurement time was 60 seconds.

In order to determine the emission efficiency of the specially designed dye-labeled gene probes and their kinetics parameters, we measured the fluorescence auto-correlation functions, and the count rates, of Rhodamine Green-labeled and Cy5-labeled gene probes in separate solution using FCS (Figure 3). The photon count rates of the two gene probes, as shown in Table 2, are high enough to ensure the reliable detection of GMOs at the single DNA molecule level by FCCS.

**Table 2:** Results for the measurement of dye-labeled gene probes by fluorescence autocorrelation spectroscopy.

Dye-Labeled Gene Probe	Laser line (nm)	τ*_diff_* § (µsec)	*N_ac_* §	*N_c_* (kHz)	Concentration (nM)
Rhodamine Green-labeled probe	488	47	3.952	10.787	41.29
Cy5-labeled probe	633	97	0.178	1.739	0.53

§ τ*_diff_* and *N_ac_* are defined in Equations (1) and (2) (see text).

**Figure 4:**
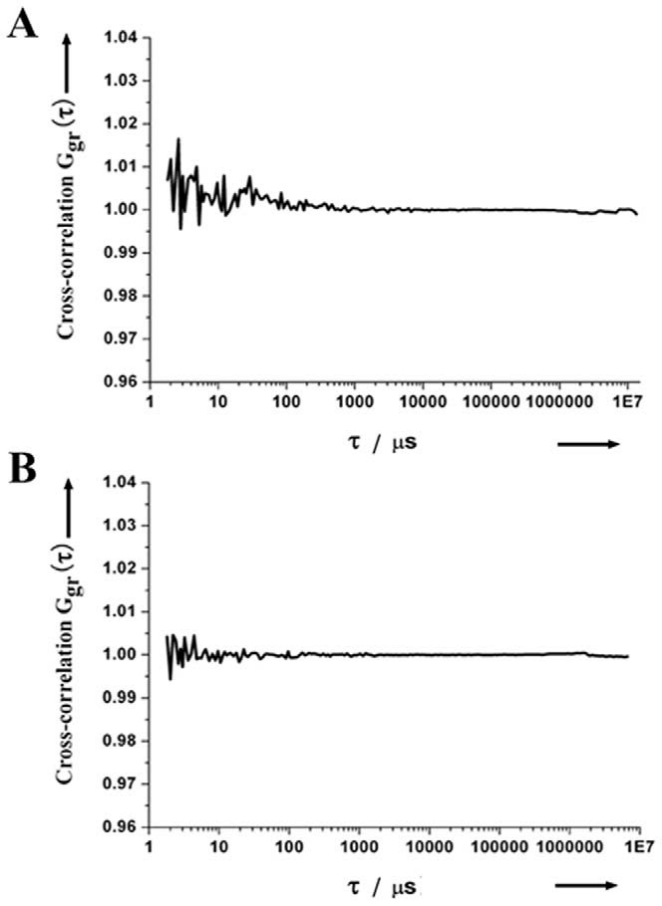
Cross-correlation functions of singly labeled gene probes. (A) RG-labeled gene probe, excited by the 488 nm laser beam. (B) Non-hybridized RG-labeled and Cy5-labeled gene probes, excited simultaneously by 488 nm and 633 nm laser beams.

To examine the proper alignment of the system and the potential cross-talking between the red and green channels, we analyzed the cross-correlation functions of singly labeled gene probes. As expected, there was no correlation between the fluorescence emission intensities detected in the red and green channels when RG-labeled gene probes were excited by the 488 nm laser beam. Similarly, no correlation was detected when physical mixture of the two dye-labeled gene probes was excited by both 488 nm and 633 nm laser beams. These results show that the cross-talking between the two channels are negligible in our measurements (Figure 4).

**Figure 5:**
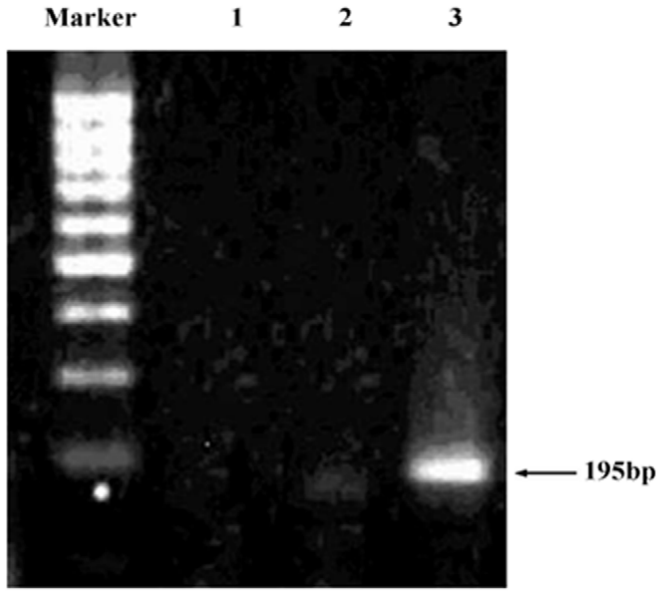
Electrophoresis detection of PCR amplified GMO in tomato samples. Left column: the DNA marker; Column 1: negative control; Column 2: non-GM tomato sample; and Column 3: GM tomato sample.

**Figure 6:**
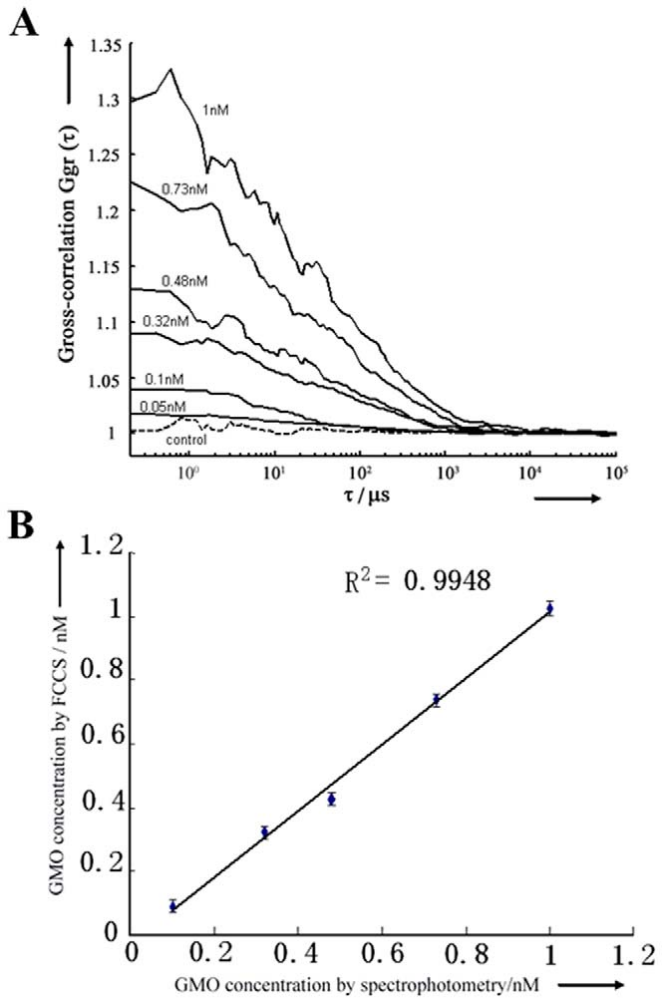
Sensitivity and reliability assessment. (A) Cross-correlation functions of PCR amplified target 195 bp fragments in GM tomatoes of different concentrations (the solid curve) and non-GM tomatoes control (the dash curve); (B) Correlation of PCR amplified GMO concentrations determined by FCCS and by spectrophotometry method at 260 nm.

Sensitivity and accuracy of our FCCS system were evaluated by detecting PCR amplified GMOs. A pair of special primers, 5′ GCTCCTACAAATGCCATCA 3′ and 5′ GATAGTGGGATTGTGCGTCA 3′, was used to amplify a 195 bp fragment in CaMV35S of GM tomato [5]. The detection of GMO using PCR amplification and gel electrophoresis shows a band of the target 195 bp fragment in the CaMV35S only in the lane of the GM tomato samples, while no PCR amplification of the 195 bp fragments was detected in the negative control and non-GM tomato samples (Figure 5). The PCR products were purified by PCR purification kit and the concentration of GMOs target was identified by spectrophotometry method. Dilution of clean PCR products was made at concentrations of 1.00 nM, 0.73 nM, 0.48 nM, 0.32 nM, 0.10 nM, and 0.05 nM. Then the products were heated for denaturation and hybridized with Cy5 and RG labeled probes, and detected by FCCS. Figure 6A shows that *G_gr_(0)* becomes larger with a higher GMO concentration of the sample. A sensitivity of about 0.05 nM was observed. Moreover, strong correlation was observed between FCCS-determined concentration and PCR standard concentration, indicating the remarkable reliability of the FCCS method (Figure 6B).

**Figure 7:**
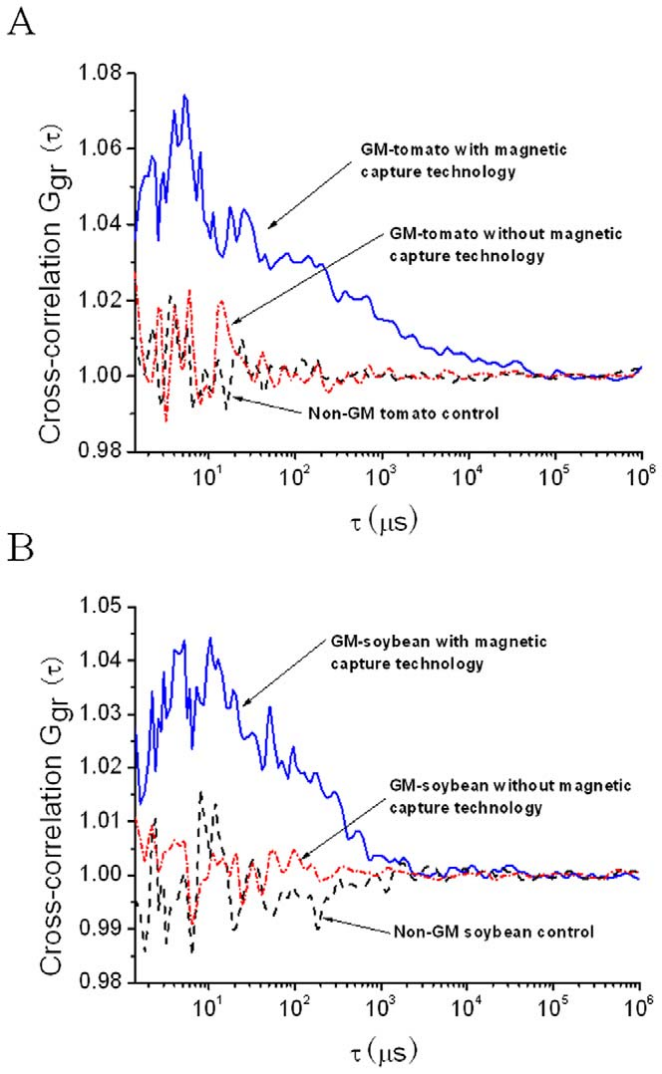
FCCS detection without PCR. Measured cross-correlation functions of the GMOs (the blue curve), non-GMOs (the black curve) with magnetic bead based samples treatments, and GMOs without magnetic bead based samples treatments (the red curve). (A) GM soybean and (B) GM tomato.

After the initial evaluation of the probes and detection parameters of current FCCS system, experiments were conducted to reach the final aim of PCR-free identification of GMOs. Two genetically modified samples (GM soybean and GM tomato) and the control samples (Non-GM soybean and Non-GM tomato) are used for the assessment of the proposed method. Genomic DNA (5 µg) from GM soybean and GM tomato was fragmented and subjected to magnetic capture pretreatment and hybridized with the two dye labeled probes. Additional experiments were also executed for directly hybrid with these two dye labeled probes to the target without magnetic capture pretreatment. For negative control experiments, genomic DNA (5 µg) from non-GM soybean and non-GM tomato was handled through a magnetic capture pretreatment procedure which was identical to the positive GM samples assay procedure. An example of GMO detection is shown in Figure 7. It was found that the magnetic capture pretreatment applied to the negative samples (non-GM soybean and non-GM tomato control) resulted in correlation amplitudes close to 1. Their fluctuations at small correlation times were due to the wide emission spectra of RG and Cy5. However, these fluctuations were not high enough to affect the outcome of GMO detection. GM soybean and GM tomato samples with the absence of magnetic capture pretreatment resulted in signal levels comparable to the signals generated by the negative controls. This is probably because of the low target concentration, or the low hybridization efficiency due to cularization of the long double strand genomic DNA and supercoiling emergence during the denaturation. In comparison, high-levels of cross-correlation were observed for both GM soybean and GM tomato samples with the magnetic capture pretreatment (Figure 7). These results demonstrated the feasibility of FCCS-based sensing strategy combined with the magnetic capture technique in detecting 35S target directly in non-amplified genomic DNA.

In this study we provided the evidence as a proof-of-concept for the FCCS-magnetic capture combination. To further clarify the characteristics of the current PCR free method for determination of GMOs, the analytical performance of the proposed assay have been compared with those of other conventional techniques, such as, ELISA [35], [36], southern blot [37], [38], PCR [39], [40], and real time PCR [41]–[43]. The major characteristics of different methods are summarized in table 3. As shown in the table, the overall performance of the current assay is better than other conventional techniques, especially the specificity. Its high specificity is the result of the selective magnetic capture of the 35S target and the hybridization of two detection probes to the same 35S region. The sensitivity of current assay (µg genomic levels) is comparable with the other PCR-free technique, southern blot. However, in this study GMOs detection takes only hours (as opposed to days for conventional southern blot method). Although the protein based ELISA is a rapid method, one of the major disadvantages is the protein denaturation as a consequence of processing. Compared to conventional PCR based method (PCR and real time PCR), which may introduce ambiguities resulting from the potential false-positive identification caused by non-specific amplifications, this PCR free method represents a significant improvement in assay reliability. Although the sensitivity of the current assay was lower than the PCR-based method, it could be improved by several orders of magnitude using a simple probe quench method, as described in the literatures [21], [23]–[24], or by enhancing the quantum yield with bi-labeling of the probe [22], or by using the quantum dot label [44]. FCCS can be easily combined with high-throughput screening, thus increasing the speed of detection as well as the number of analyzed samples [45]. The combination of magnetic capture technology with FCCS presented in this study should not be restricted only to the detection of unamplified GMOs samples. The strategy and the procedures can be easily adapted for assaying other specific nucleic acids.

**Table 3:** Comparison between the current method and other conventional techniques for GMOs detection.

Parameter	ELISA	Southern blot	PCR	Real time PCR	Current assay
Sensitivity	Moderate	Moderate	High	High	Moderate
Specificity	Fair	Good	Fair	Fair	Excellent
Assay time	6–8 h	2–4 d	1.5 d	1 d	8–10 h
Potential to be quantitative	Yes	No	No	Yes	Yes
Needs special equipment	Yes	Yes	Yes	Yes	Yes
